# Development of Anti-idiotypic Monoclonal Antibody Mimicking SARS-CoV-2 Receptor Binding Domain

**DOI:** 10.1007/s12033-024-01138-1

**Published:** 2024-04-25

**Authors:** Gamze Kılıç, Elif Demirkan, Fatıma Yücel

**Affiliations:** 1 Bursa Uludag University, Faculty of Arts and Sciences, Biology Department, Görükle Campus, Bursa, Turkey; 2 TUBITAK, Marmara Research Center, Life Sciences, Genetic Engineering and Biotechnology, Kocaeli, Turkey

**Keywords:** Severe Acute Respiratory Syndrome Coronavirus 2, Anti-idiotypic antibody, Vaccine, Fragment antigen-binding, Monoclonal antibody, Hybridoma

## Abstract

Using the hybridoma technique, we developed a panel of anti-idiotypic monoclonal antibodies (aId-mAb) that mimic The Severe Acute Respiratory Syndrome Coronavirus 2 (SARS-CoV-2) Receptor-Binding Domain (RBD) molecule against Fragment antigen-binding (Fab) of anti-SARS-CoV-2 (S1, RBD) antibodies. Investigated the in vivo and in vitro effects of these aId-mAbs we developed and examined their antigenic mimicry abilities. Among these 12 antibodies, 6 aId-mAbs (designated FY1B4, FY2A6, H9F3, E6G7, FY7E11, and FY8H3) were selected for further characterization in a series of experiments. First, competitive receptor binding assay results confirmed that six aId-mAbs could specifically bind to the ACE2 receptor in target cells and block the interaction between the RBD molecule and the ACE receptor. Moreover, we examined the immunological activities of these aId-mAbs in female BALB/c and showed that E6G7, H7E11, and H8H3 aId-mAbs induce an antibody response by mimicking RBD and stimulating the immune system. It is considered that these three aId-mAbs will be evaluated as SARS-CoV-2 vaccine candidate molecules in future studies.

## Introduction

SARS-CoV-2 virus, the causative agent of coronavirus disease (COVID-19), is a new strain discovered in December 2019 and not previously identified in humans. SARS-CoV-2 has been identified as a new beta coronavirus strain of the Coronoviridae family [1].

The binding of the SARS-CoV-2 virus to the cell occurs through the interaction between the Spike (S) protein of the virus and the Angiotensin-converting enzyme 2 (ACE2) in the host cell. The S glycoprotein of SARS-CoV-2 binds to cellular ACE2 [2–4] and initiates viral–host membrane fusion, thereby permitting viral entry into the host cell [5, 6]. S protein has a Receptor-Binding Domain (RBD) in the S1 region, and the RBD domains vary depending on the virus [7–9]. Interaction with the receptor is vital for the S protein to interact with the host. The S proteins of coronaviruses are found on the surface of the virus. Because they interact with the host cell, they are the focus of therapeutic vaccine design and the main target of neutralizing antibodies [10]. Antibodies with neutralizing effects are critical immune system molecules in viral diseases [11]. Monoclonal antibodies (mAbs) targeting the S protein on the surface of coronaviruses are becoming increasingly crucial as promising drugs for disease treatment. Targeted neutralizing antibodies can neutralize RBD and prevent it from binding to the ACE2 receptor. Therefore, the use of neutralizing mAbs in therapeutic studies has become widespread. In addition, it has been shown in experimental studies that aId-mAbs developed using mAbs with neutralizing effects provide active and passive protection [12]. The variable regions of the antibody molecule that recognize the antigen are called idiotypes (Id). AId-mAbs can trigger antibody formation by mimicking the binding sequences of the antigen [13, 14]. AId-mAbs act as copies of the original antigen, and because of these properties, they are promising as an alternative method in vaccine studies without causing disease. Many studies are in the literature to obtain the Fab region where these Id parts of the antibody molecule are found [15]. AId-mAbs mimic and act as copies of the antigenic molecule (RBD) that initiates the disease process. When presented to the immune system in this state, they form antibodies against the antigen they mimic. These molecules can be considered vaccine candidate molecules that mimic VLPs, or fundamental virus particles, in virus-like particle (VLP) vaccines.

In this work, A panel of aId-mAb that mimic the SARS-CoV-2 RBD molecule against Fab of anti-SARS-CoV-2 (S1, RBD) mAbs was created by us using the hybridoma process. Investigated the effects of these aId-mAbs that we developed both in vivo and in vitro, as well as their capacity for antigenic mimicry.

## Material and Method

### Reagents and Materials

This study used two murine mAbs (shown that were a neutralizing antibody and a non-neutralizing antibody) targeting the SARS-CoV-2 spike protein (S1) that were generated in a previous project, as well as a human antibody purified from the convalescent plasma of a SARS-CoV-2-infected human donor (shown that a neutralizing antibody) (TÜBİTAK, Gebze, Koceli, Turkey). Frozen hybridoma cells producing anti-SARS-CoV-2 (S1 protein) mouse mAbs were cultured. Immunoaffinity chromatography with solid phase-linked protein G (Mab-Trap/Pharmacia) (Marlborough, Massachusetts) was used to purify the antibodies in the supernatants. Most chemical reagents were obtained from Sigma Aldrich (St. Louis, Missouri, USA).

The myeloma cell line F0 (ATCC CRL-1646, (www.atcc.org)) was used for in vitro studies. The experimental animals used in this study were obtained from the TÜBİTAK (Gebze, Kocaeli, Turkey) experimental animals’ unit (TÜBİTAK HADYEK Ethics Committee No. 16563500-111-64-3028).

### Enzymatic Digestion of Antibodies with Papain

Human polyclonal antibodies (pAbs) and two mouse mAbs obtained from the serum of patients with prior COVID-19 were digested with papain at a ratio of 1/10, 1/20, and 1/50 (enzyme/antibody; v/v), and Fab and Fc regions were obtained. Fragments were separated on a protein column, and regions of pure Fab were checked by SDS-PAGE, silver staining, and Western blotting.

According to the protocol for digesting, papain was dissolved in water. It was then added to an activation buffer containing 50 mM l-cysteine, 20 mM NaH_2_PO_4_, 10 mM EDTA, and a pH of 7. The mixture was passed through a vivaspin 500 column in papain digestion buffer (20 mM NaH_2_PO_4_, 10 mM EDTA, pH 6.3). Various percentages of antibodies were combined with papain. After digestion, with SDS-PAGE shown the Fab and Fc regions of the antibody, these antibodies were metabolized in a large volume. The column was connected to an NGC (such as New Generation Chromatography) system to be purified. Washing was done with binding buffer (3 M NaCl, 1.5 M Glycine pH: 8.5) up to five times (5×) the column volume. Then, the digested antibodies were loaded into the appropriate unit in the NGC system for delivery to the protein in an affinity column. A binding buffer was passed through the system, and post-column fractions were collected in 1 ml tubes. Fab regions not binding to protein A were collected in the pre-cut. Elution buffer (0.1 M Glycine–HCl pH: 2.7) was passed through the column. Antibodies were collected in 1 ml tubes containing 1 M Tris–HCl, pH 9 equilibration buffer. The Fc regions and the non-digested antibodies were obtained in the elution part. Then, to confirm the purity, the fragments run by SDS-PAGE were shown by silver staining, and the fractions containing pure Fab regions were combined and dialyzed.

### Immunization of Mice with the Fab Region

Fab regions of anti-SARS-CoV-2 murine mAbs (anti-S1 MAM H9 and anti-S1/RBD MAM E6 mAbs) and human anti-SARS-CoV-2 polyclonal antibodies (anti-S1 hAb) were obtained after enzymatic digestion. Three immunization groups were created for three Fab regions with 8-week-old female BALB/c. Mice were inoculated intraperitoneally every 14 days with 50 µg of the fab region of each antibody. Three antibody fragments were emulsified with equal volumes of Freund’s complete or incomplete mineral oil adjuvant. Booster immunizations (Rappel) were administered systemically in phosphate-buffered saline (PBS). On the tenth day after immunization, a small volume of serum was collected from mice, and antibody responses to antigens were determined using indirect ELISA.

### Indirect ELISA

Utilizing indirect ELISA, the immune response and hybridoma supernatant in the serum of mice were screened. Enzyme immunoassay plates (Nunc, Roskilde, Denmark) were coated with 100 µl of ACE2 protein per well at 150 ng/ml concentration in PBS buffer (pH: 7.2) overnight at 4 °C. The plate was washed three times with 0.05% (v/v) PBS-Tween-20 (PBS-T). Non-specific bindings were blocked with 1.5% skim milk (SMP) for 1 h at 37 °C and washed three times with PBS-T. Mouse serum or hybridoma supernatant was incubated for 1 h at 37 °C with S1 immune mouse serum as the positive control. After washing the plate, antibodies were detected for 1 h at 37 °C with alkaline phosphatase-conjugated goat anti-mouse polyvalent (IgG, IgM, IgA) diluted 1/2000 in PBS buffer. The plate was washed five times with PBS-T. Finally, *Para*-nitro phenyl phosphate (1 mg/ml solution in substrate buffer) was added, and the reaction was measured using an enzyme immunoassay (EIA) reader equipped with a Bio-Tech Synergy 2 enzyme immunoassay (EIA) plate reader at 405 nm absorbance.

### Cell Fusion and mAb Production by Hybridoma

Cell fusion and mAb production were completed by modifying the Köhler and Milstein method [16]. The myeloma cell’s immortality and the B cell’s ability to produce antibodies has been combined into a single hybridoma cell as a result of the fusion study. Lymphocytes from the spleen and lymph nodes (bronchial, axillary, inguinal, popliteal, and intraperitoneal) were fused with F0 (ATCC CRL 1646) mouse myeloma cells.

Fifty percent polyethylene glycol 4000 (PEG, Roskilde, Denmark) was used as the coupling agent and after fusion, the cell mixture was resuspended in DMEM containing hypoxanthine aminopterin thymidine (HAT; Gibco, Waltham, MA), 20% fetal calf serum and antibiotics. Plated in 96-well plates and incubated overnight at 37 °C, 5% CO_2_, and 95% humidity.

Ten to fifteen days after fusion, the wells were screened for antibody selection determined by indirect ELISA. Positive wells were cloned using the limiting dilution method, and macrophages were used as feeder cells.

### Determination of Antibody Subclasses

Class and subclass of mAbs were determined using a BD Mouse Immunoglobulin Isotyping ELISA Kit (East Rutherford, New Jersey, USA) purchased from Fisher Scientific. Following the established methodology, each monoclonal antibody specific to a specific isotype of rats and purified for mice was used to coat the wells of an ELISA plate. The ELISA plate washed off three times with PBS-T. Subsequently, a blocking buffer was added to each well. Following incubation and washing, the hybridoma culture supernatant was added onto the plate, followed by the addition of a solution containing HRP-labeled rat anti-mouse Ig mAb. Each well was analyzed by measuring the absorbance at 450 nm using the HRP substrate.

### Purification and Characterization of Antibodies

Monoclonal antibodies were purified from the hybridoma supernatant by precipitation of ammonium sulfate ((NH_4_)_2_SO_4_) (A4418, Sigma Aldrich) at 30–50% saturation. For the precipitation of mAbs from the culture supernatant, 341 g/l of crystalline (NH_4_)_2_SO_4_ was added gently with agitation and left at 4 °C overnight for protein precipitation. The mixture was centrifuged at 13,000 rpm for 1 h at 4 °C, and the particle was resuspended in 10 ml of PBS and dialyzed for 24 h against 100 times the volume of PBS at 4 °C. Using HiTrap Protein a (*Staphylococcus aureus*), (Cytiva, Switzerland), antibodies were purified by immunoaffinity chromatography. All aId-mAbs were characterized for cross-reactivity with human serum, ACE2, RBD, S1, NP, and PBS using indirect ELISA.

### Labeling of ACE2 with Biotin

In biotin labeling studies, an amine-specific biotinylated agent, biotin *N*-hydroxyl-succinimide ester (NHS-Biotin; Sigma, USA), was dissolved in DMSO. Before adding biotin, the pH of the ACE2 protein solution was adjusted to 9 with 0.1 M carbonate buffer. 1 mg of ACE2 protein and 125 µg of biotin were combined and incubated at room temperature for 4 h. By overnight dialyzing against PBS, unreacted biotin was removed from the mixture.

### ACE2 Neutralization Test (w/ELISA)

A competitive ELISA assay was used to identify aId-mAbs that mimic the RBD protein. For this, 96-well microtiter polystyrene ELISA plates (Nunc, MA) were coated with 250 ng of RBD in PBS buffer and incubated overnight at +4 °C. The plate was washed three times with PBS-Tween 20.

The ELISA plate was incubated with 2% milk powder for 1 h at 37 °C. The wells were then washed three times with PBS-T. Different concentrations of aId-mAb (250, 500, 100 ng) were incubated with a fixed amount of biotin-labeled ACE2 (1/250 diluted) in separate tubes. After that, mixtures of aId-mAb and biotin-labeled ACE2 were dispensed into ELISA plate wells. After 30 min of room temperature incubation, the plate was washed three times with PBS-T. The plate wells were filled with 100 µl of streptavidin-pod conjugate buffer and incubated at 37 °C for 30 min in the dark. The ELISA plate was washed off five times and incubated for 10 min with TMB substrate buffer. The reaction was terminated by adding H_2_SO_4_ and measured at 450 nm.

### Mouse Immunization with aId-mAbs

Six of the 12 aId-mAbs were selected, and seven immunization groups were created using the RBD molecule as the control group. 50 µg aId-mAb and 2.5 µg RBD protein per mouse were diluted in 100 µl PBS mixed with 100 µl Freund’s adjuvant and immunized intraperitoneally at 2-week intervals. A small amount of serum was collected from mice on the seventh day after immunization, and antibody responses to antigens were determined by indirect ELISA.

## Results

### Digestion of Antibodies with Papain

All three anti-SARS CoV-2 antibodies were digested using the enzyme papain. Fab regions were then obtained in pure form.

**Fig. 1:**
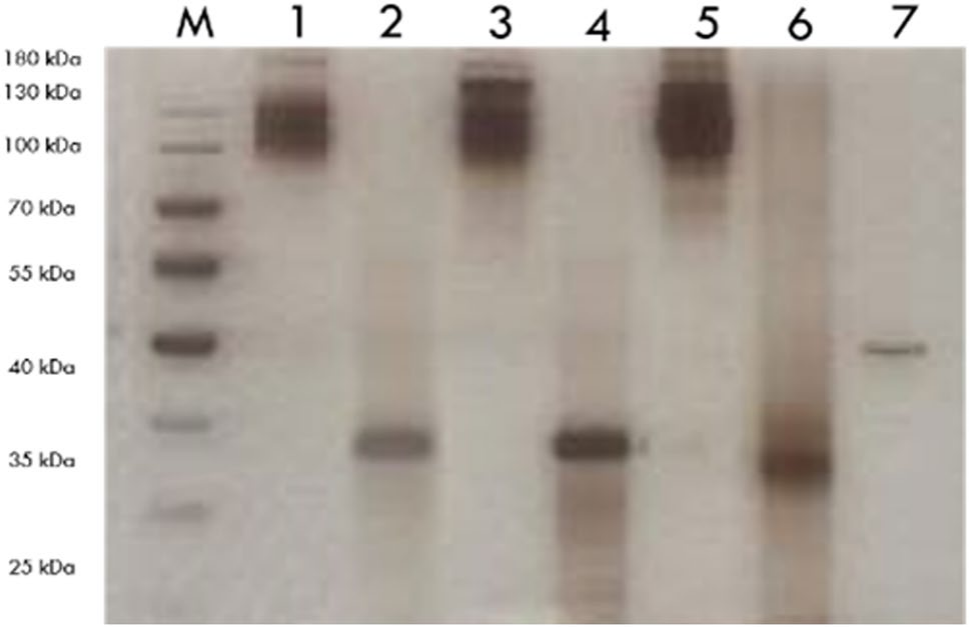
Silver-Stained gel to show Monoclonal antibodies and Fab regions. M, Marker (Thermo Fisher); 1, Mouse anti-S1 MAM H9 monoclonal antibody; 2, Mouse anti-S1 MAM H9 fragment antigen-binding; 3, Mouse anti-S1/RBD MAM E6 monoclonal antibody; 4, Mouse anti-S1/RBD MAM E6 fragment antigen-binding; 5, Human anti-S1 FY polyclonal antibody; 6, Human anti-S1 FY human fragment antigen-binding; 7, Bovine Serum Albumin for control (A2153, Sigma Aldrich)

**Fig. 2:**
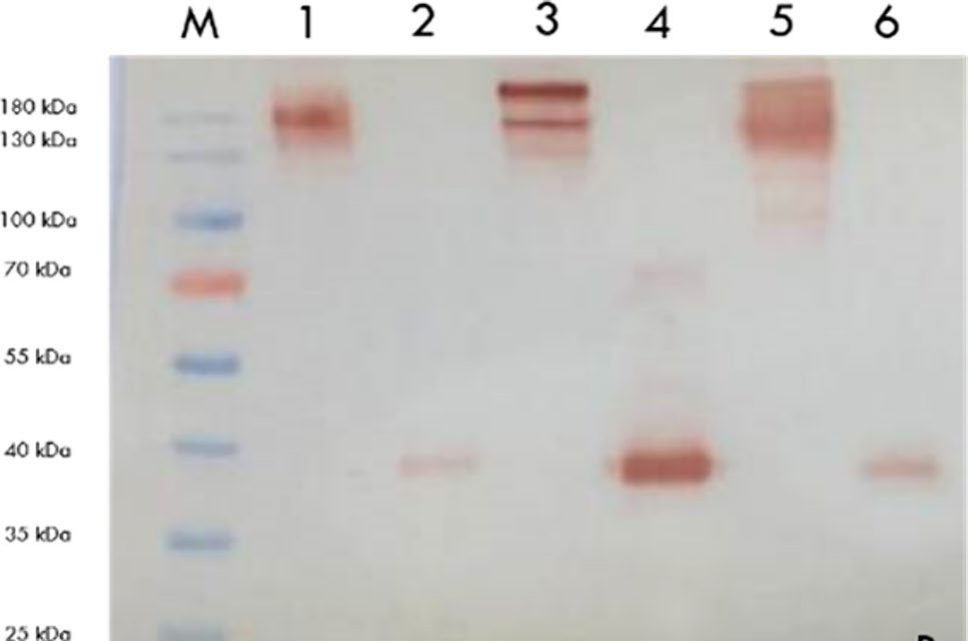
Western blot to show Monoclonal antibodies and Fab regions. M, Marker (Thermo Fisher); 1, Mouse anti-S1 MAM H9 monoclonal antibody; 2, Mouse anti-S1 MAM H9 fragment antigen-binding; 3, Mouse anti-S1/RBD MAM E6 monoclonal antibody; 4, Mouse anti-S1/RBD MAM E6 fragment antigen-binding; 5, Human anti-S1 FY polyclonal antibody; 6, Human anti-S1 FY human fragment antigen-binding

The purity of the Fab regions obtained after the proteolytic enzyme digestion of the antibodies was demonstrated by SDS Page and silver staining (Fig. 1). It was shown by Western blot analysis that antigen responses of epitopes in the idiotypic domain in the Fab regions continued after the enzyme digestion of the antibodies (Fig. 2).

Immunization of mice was initiated after naive Fab regions continued to elicit an antigenic response with the SARS-CoV-2 S1 protein.

### Generation of aId-mAbs

**Fig. 3:**
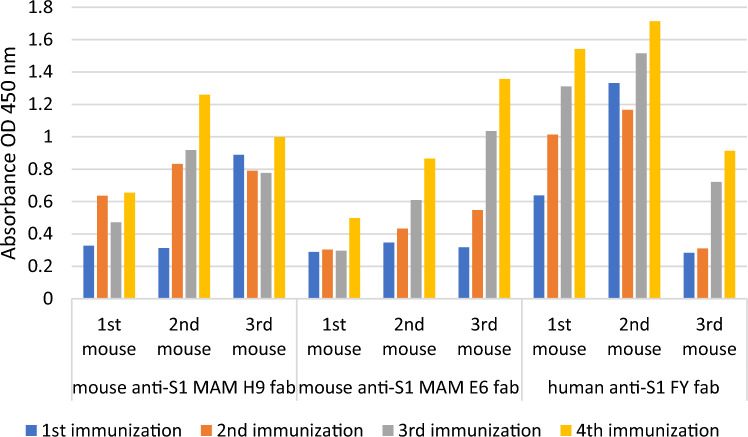
Immune responses of mice injected by mouse anti-S1 MAM H9 monoclonal antibody, mouse anti-S1/RBD MAM E6 monoclonal antibody, and human anti-S1 FY polyclonal antibody

The immunization study was started using three Fab regions with nine mice in three different cages separately. Blood sera obtained following immunizations were tested against ACE2 protein by indirect ELISA (Fig. 3).

Immune response have tested by indirect ELISA. To test the immune responses of mice, blood sera were collected on the tenth day after each immunization. With PBS diluted 1/5000 times, it underwent indirect ELISA testing in antigen-coated wells. Fusion studies were performed, respectively, with mice whose response at OD 450 nm was <1.00 as a result of ELISA. Fusion studies were performed with mice that showed a high antibody response after the fourth immunization.

After three fusions, 22 mAbs were developed that produced hybridoma cells. Three of these hybrid cells lost their antibody activity. To determine the affinities of the remaining 19 mAbs, varying concentrations of ACE2 were plated into ELISA wells. It was also tested in SP, RBD, NP, human serum, and antigen-free wells to show whether the antigen response of the antibodies was specific. Six antibodies were determined to be non-antigen specific (data not shown). The remaining 13 mAbs were shown to recognize ACE2 specifically. Two aId-mAbs (H9F3, H9F10) were obtained from the fusion of mice immunized with anti-S1 H9-fab, and one aId-mAb (E6G7) was obtained from the fusion of mice immunized with anti-S1/RBD E6-fab.

**Fig. 4:**
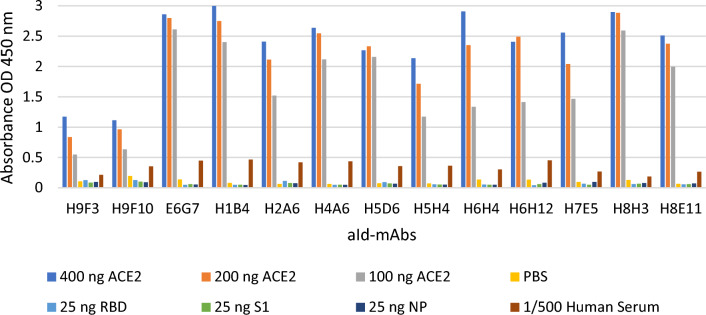
Crossover ELISA of aId-mAbs

In addition, ten aId-mAbs were developed from a fusion of mice immunized with human anti-S1 FY-fab (H1B4, H2A6, H4A6, H5D6, H5H4, H6H4, H6H12, H7E5, H8H3, H8E1 (Fig. 4).

Immunoglobulin sub-isotypes for the 13 mAb were determined with the ELISA kit (BD Pharmingen™, 550487). The sub-isotype information was determined according to the ELISA results (Table 1). For each type of immunoglobulin, a suitable purification process was used.

**Table 1:** Determination of sub isotypes of mAbs

Antibody	Sub-isotype	Chain
H9F3	IgG3	(Kappa chain)
H9F10	IgG1	(Kappa chain)
E6G7	IgG2a	(Kappa chain)
H1B4	IgG1	(Kappa chain)
H2A6	IgG2b	(Kappa chain)
H4A6	IgG1	(Kappa chain)
H5D6	IgG1	(Kappa chain)
H5H4	IgG2b	(Kappa chain)
H6H4	IgG3	(Kappa chain)
H6H12	IgM	(Kappa chain)
H7E5	IgG2a	(Kappa chain)
H8H3	IgG2a	(Kappa chain)
H8E11	IgG1	(Kappa chain)

Mammals classified antibodies into five primary classes or isotypes—IgA, IgD, IgE, IgG, and IgM. The IgG class in mice is categorized into five sub-classes: IgG1, IgG2A, IgG2B, IgG2C, and IgG3. AId-mAbs that were produced were classified as five IgG1, three IgG2a, two IgG2b, two IgG3, and one IgM. The aId-mAbs were purified using column chromatography based on their sub isotype.

### Demonstration of In Vivo and In Vitro Effects of aId-mAbs

The ability of aId-mAbs to elicit a response to anti-RBD antibodies and mimic antigens was investigated in both ELISA and BALB/c. By competitive ELISA, the ability of the developed 13 aId-mAbs to inhibit binding to the RBD protein coated in the ELISA plaque well after interacting with biotin-labeled ACE2 was tested.

Pure mouse mAbs (250, 500 and 1000 ng/well) were individually pre-incubated with Biotin-labeled ACE2 (1/750-fold diluted) and tested by adding them to RBD-coated ELISA plate wells. The results of competitive ELISA testing performed in wells coated with 100 ng of RBD were assessed.

PBS was a negative control in the study; Commercial RBD molecule was used as a positive control. According to the experimental results, the ability of the antibodies to neutralize the RBD protein in a 200 ng ACE2 coated ELISA well was determined (Table 2).

**Table 2:** Neutralization percentages of antibodies

Sample	Neutralization %	Sample	Neutralization %
PBS	0.00	H9F3	65.13
H9F10	2.32	H2A6	67.86
H4A6	7.05	H8H3	68.23
H5D6	8.52	H1B4	69.74
H8E11	8.75	H7E5	75.46
H5H4	9.52	E6G7	77.08
H6H4	22.10	RBD	92.21
H6H12	29.23		

Of the 12 aId-mAbs tested, 6 were shown to block the RBD binding site on the ACE2 surface at higher percentages than the others (E6G7, H7E5, H1B4, H8H3, H2A6, H9F3 aId-mAbs). Mice immunizations were initiated with these six antibodies, and the in vivo effects of aId-mAbs were investigated. As the positive control of the study, an immunization group with a commercial RBD mixture (delta strain, Wuhan strain, and England strain) was formed. Six of these antibodies with the highest ACE2 response were selected. Six different immunization groups were created with six different antibodies. Three mice were immunized in each group. 50 µg of Fab region was used as the immunization dose. Group 7th, inoculated with 4 µg RBD mix proteins, was formed to be the experiment’s control group. RBD antigen responses of mice were checked by performing indirect ELISA with blood sera from mice.

**Fig. 5:**
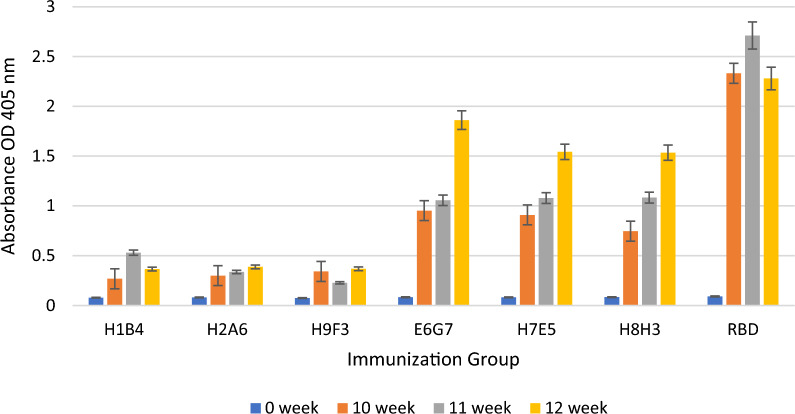
Determination of the immune responses against the RBD of the UK strain by ELISA after immunization with aId-mAbs

**Fig. 6:**
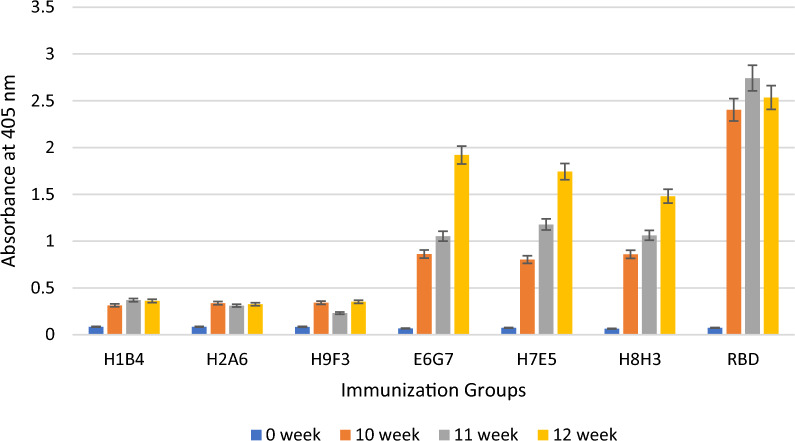
Determination of the immune responses against the RBD of the Wuhan strain by ELISA after immunization with aId-mAbs

**Fig. 7:**
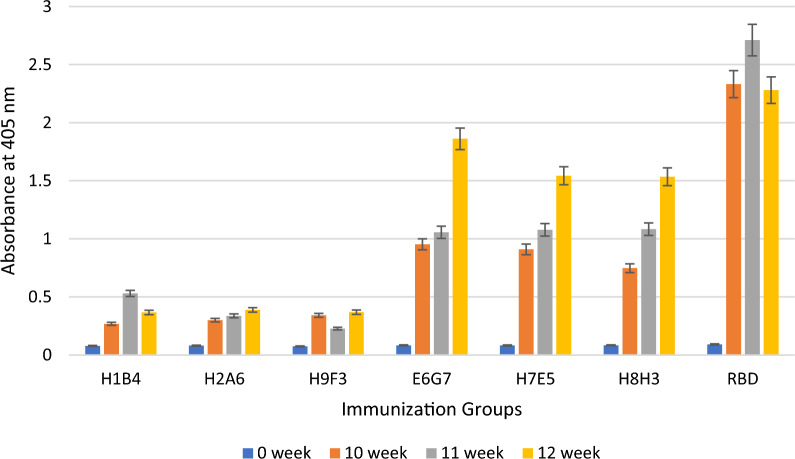
Determination of the immune responses against the RBD of the Delta strain by ELISA after immunization with aId-mAbs

Antibody responses of mice were tested in RBD coated ELISA wells with serum of mice collected after the fifth, sixth, and seventh immunizations. Three different variants of SARS-CoV-2/RBD (Delta, Wuhan, and UK form) were used as coating antigens. Blood sera from immunized mice were tested with the UK (Fig. 5), Wuhan (Fig. 6), and Delta strains (Fig. 7). Blood serums were tested by diluting 500 times.

The bar graph illustrates the average values of the antibody responses obtained from the mice in each immunization group at week 0 (before to immunization), week 10 (following the fifth immunization), week 11 (following the sixth immunization), and week 12 (following the seventh immunization). As seen in the graphics, in-vivo studies show that aId-E6G7, aId-H7E5, and aId-H8H3 antibodies can mimic the RBD molecule compared to mice immunized with the RBD molecule.

In conclusion, using hybridoma technology, aId-mAbs that mimic RBD antigens were developed and demonstrated in vivo to mimic commercial RBD antigens.

## Discussion

The development of the SARS-CoV-2 vaccine in 2020 surpassed all previous vaccine development studies and spawned over 300 vaccine development initiatives in a matter of months [17]. In this process, approved vaccines for SARS-CoV-2 inactivated vaccines (Sinopharm and SinoVac), vector-based vaccines (CanSinoBio, Sputnik V, and AstraZeneca), and mRNA-based vaccines (Pfizer BioNTech and Moderna) [18] were launched. In addition to all these methods, aId vaccines, which have been prepared against bacterial and viral pathogens and are effective, may also constitute an alternative approach for SARS-CoV-2. AId-mAbs can bind to the idiotypic variable domains of an antibody [19]. In this way, aIds can trigger antibody formation [20] by mimicking the binding sequences of antigens [21]. Using aId-mAbs as a vaccine provides the development of antibodies with high protection against the epitope it mimics [22].

Polyclonal and monoclonal idiotypes were used comparatively in this study. In this context, unneutralizing mouse mAb (MAM-H9) and neutralizing mouse mAb (MAM-E6) targeting the anti-SARS-CoV-2 spike protein (S1/RBD) and anti-SARS-CoV-2 neutralizing Human pAbs were used. Three antibodies were cut with papain enzyme separately; Fab regions of the antibodies were purified and used to immunize BALB/c. After immunization, mice with the highest antigenic response to the RBD receptor ACE2 were fused, respectively. B-lymphocytes of these mice were isolated and combined with myeloma cells.

A faster antigenic response was observed among the mouse groups created in mice immunized with polyclonal idiotypes. As a result of fusion studies with mice immunized with both mouse monoclonals and human polyclonal idiotypes, when the number of specific hybridoma cells obtained for the target molecule was compared, it was shown that the efficiency was much higher in mice immunized with polyclonal idiotypes. This may be due to the increase in the overall avidity of the target due to multiple epitope bindings [23].

Indirect evidence that the cellular immune response develops after immunization studies is IgG2a type antibodies. IgG2a and IgG1 antibodies, in particular, through opsonization, complement fixation, and immunological effector activities, are crucial for pathogen protection [24].

Twelve aId-mAbs were generated in this study. The six generated aId-mAbs (H9F3, H2A6, H8H3, H1B4, H7E5, and E6G7) have shown competitive binding with RBD by ACE2 neutralizing ELISA. The demonstrated neutralizing capacity exceeds 65%. Nevertheless, three aId-mAbs (E6G7, H7E5, and H8H3) have been shown to induce an anti-RBD immune response through in vivo mouse immunizations. Of these three aId-mAbs, two have been obtained from human neutralizing SARS-CoV-2 mAb and one has been obtained from mice immunized with mouse neutralizing SARS-CoV-2 mAb. Of the three aId-mAbs that create an anti-RBD response after in vivo immunizations, the antibody sub isotypes frequently seen in aId-mAb development studies are IgG2a, as defined in the literature.

It has been clearly shown that the developed aId-mAbs create an immune response following competitive neutralizing ELISA testing with ACE2 protein and in vivo mouse immunizations. Thus, it has been shown that the aId-mAbs obtained from the study can be used as copies of the original SARS-CoV-2 Surface antigen and can be evaluated as an alternative to commercial vaccines. Large-scale production of hybridomas expressing aId-Fab antibodies in cell culture systems will enable abundant SARS-CoV-2 surface antigens to be obtained safely and economically. This study is only a preliminary study for developing a vaccine-candidate molecule. The aId-mAbs developed in the study have been shown to mimic RBD, the SARS-CoV-2 surface antigen. Challenge experiments will demonstrate their protective effectiveness in the follow-up study.

## Data Availability

Data supporting the findings of this study are available from the corresponding author upon reasonable request. The data are not publicly available due to privacy or ethical restrictions.
